# Central hypersomnia and chronic insomnia: expanding the spectrum of sleep disorders in long COVID syndrome - a prospective cohort study

**DOI:** 10.1186/s12883-022-02940-7

**Published:** 2022-11-09

**Authors:** Alissa Elen Formiga Moura, Danilo Nunes Oliveira, Danielle Mesquista Torres, José Wagner Leonel Tavares-Júnior, Paulo Ribeiro Nóbrega, Pedro Braga-Neto, Manoel Alves Sobreira-Neto

**Affiliations:** 1 Hospital Universitário Walter Cantídio, Neurology Service, Universidade Federal do Ceará, Fortaleza, Brazil; 2 Department of Clinical Medicine, Universidade Federal do Ceará, Rua Prof. Costa Mendes, 1608 - 4° andar - Rodolfo Teófilo, Fortaleza, Ceará Brazil; 3 Center of Health Sciences, Universidade Estadual do Ceará, Fortaleza, Brazil

**Keywords:** Long-COVID, Sleep, Hypersomnia, Narcolepsy

## Abstract

**Introduction:**

Long-onset COVID syndrome has been described in patients with COVID-19 infection with persistence of symptoms or development of sequelae beyond 4 weeks after the onset of acute symptoms, a medium- and long-term consequence of COVID-19. This syndrome can affect up to 32% of affected individuals, with symptoms of fatigue, dyspnea, chest pain, cognitive disorders, insomnia, and psychiatric disorders. The present study aimed to characterize and evaluate the prevalence of sleep symptoms in patients with long COVID syndrome.

**Methodology:**

A total of 207 patients with post-COVID symptoms were evaluated through clinical evaluation with a neurologist and specific exams in the subgroup complaining of excessive sleepiness.

**Results:**

Among 189 patients included in the long COVID sample, 48 (25.3%) had sleep-related symptoms. Insomnia was reported by 42 patients (22.2%), and excessive sleepiness (ES) was reported by 6 patients (3.17%). Four patients with ES were evaluated with polysomnography and test, multiple sleep latencies test, and actigraphic data. Two patients had a diagnosis of central hypersomnia, and one had narcolepsy. A history of steroid use was related to sleep complaints (insomnia and excessive sleepiness), whereas depression was related to excessive sleepiness. We observed a high prevalence of cognitive complaints in these patients.

**Conclusion:**

Complaints related to sleep, such as insomnia and excessive sleepiness, seem to be part of the clinical post-acute syndrome (long COVID syndrome), composing part of its clinical spectrum, relating to some clinical data.

## Introduction

The new coronavirus SARS-CoV-2 rapidly spread around the world, turning into a pandemic [1, 2]. SARS-CoV-2 enters cells through interaction with angiotensin converting enzyme-2 (ACE2) receptors present in many cell types, including nasal mucosa, lungs, heart, liver, kidneys and brain, as well as arterial and venous endothelial cells [2]. Once internalized, the virus replicates and matures, leading to a widespread inflammatory response with massive cytokine release [3].

The World Health Organization (WHO) defines “post-COVID-19” as a symptom complex with typical symptoms such as fatigue, shortness of breath and cognitive disorders, which occurs within 3 months after infection, lasts at least 2 months, can fluctuate and for which there is no explanatory explanation there is not attributed to alternative diagnoses. The English NICE (National Institute for Health and Care Excellence) guidelines distinguish between acute COVID-19 illness (< 4 weeks after infection), “persistent COVID-19 symptoms” (4–12 weeks) and post-COVID Syndrome (> 12 weeks), with the last two stages commonly grouped together as “Long-COVID” [4–7].

Long-term effects of COVID-19 occur in up to 32% of infected patients. Persistence of symptoms after active infection has also been reported in diseases caused by other coronaviruses, such as the SARS epidemic of 2003 and the 2012 MERS epidemic [8].

While at the beginning of the pandemic a large number of investigations focused on the psychosocial effects of the COVID-19 pandemic on sleep [9], there is now a wealth of data on sleep disorders as a result of SARS-CoV-2 infection. A systematic review and meta-analysis of 39 cohort studies on persistent symptoms in the long-term course after COVID-19 reports a prevalence of sleep disorders of 36% for the first 3 months and 33% for the period from 3 months to just over half a year, particularly insomnia [10–14].

The objective of this study was to assess the prevalence and characteristics of sleep-related symptoms in long COVID-19 patients in a Brazilian population, specifically analyzing the causes of excessive sleepiness.

## Methods

This was a prospective cohort study. We evaluated patients with post-COVID symptoms for the presence of sleep disorders and conducted a more extensive analysis in those with excessive sleepiness.

### Study population

A total of 207 consecutive patients with post-COVID symptoms were evaluated at a neurology outpatient clinic in Hospital Universitário Walter Cantídio – Universidade Federal do Ceará in the state of Ceará, northeast Brazil, from August 2020 to September 2021. The recruitment of patients was carried out by invitation on all local news and radio stations, as well as on the hospital’s Instagram. A number for contacting and making an appointment was provided. Patients who contacted this number were screened for enrollment in the study.

Considering an estimated prevalence of insomnia in the general population of 10%, the sample size was calculated to detect statistically significant differences with the R software (R version 4.2.1, Free Software Foundation’s GNU General Public License), using the code samplingbook::sample.size.prop(e = 0.05,*P* = 0.10,N = Inf, level = 0.95). This resulted in a total of *N* = 139 patients. After this step, we stipulated a dropout of 25% to compose the final sample, totaling a minimum sample size of 186 participants [15].

Inclusion criteria were previous infection by SARS-CoV-2 documented with RT–PCR assay (Allplex™ SARS-CoV-2 Assay - N/RdRP/S genes – Seegene Brazil), persistence of symptoms for longer than four weeks and, in patients with sleep disorders, new onset of sleep-related symptoms after COVID infection. Patients who did not fulfill the inclusion criteria or who did not agree to participate in the study were excluded.

Patient data were collected using a semi structured questionnaire. Sociodemographic data, COVID infection severity, defined by hypoxemia, systemic involvement and/or need for hospital admission, neurologic symptoms, cognitive performance and psychiatric symptoms were assessed. Psychiatric disorders were evaluated through a structured psychiatric interview conducted by a neurologist. Participants underwent application of the Geriatric Depression Scale (GDS) to assess mood, or the Beck Inventory, depending on patient age. We used a cutoff point of 3 on the GDS and 10 on the Beck inventory for the diagnosis of depression [16, 17]. Psychiatric disorders were classified according to the Diagnostic and Statistical Manual of Mental Disorders (DSM-5). Participants were submitted to the Addenbrooke’s Cognitive Examination–Revised (ACE-R) and Mini-Mental State Examination (MMSE). Cutoff values of 58, 76 and 83 points were used for patients with < 4, 4–8 and > 8 years of education, respectively, in the ACE-R [18, 19]. For the MMSE, cutoffs of 19 for illiterate patients and 24 for literate patients were used [20, 21]. Cognitive diagnosis was established according to scores on the MMSE and ACE-R.

### Subgroup analysis of excessive sleepiness (ES) patients

Excessive sleepiness is the cardinal feature of the central disorders of hypersomnolence. ES is defined as the “inability to stay awake and alert during major waking episodes of the day, resulting in periods of irrepressible need for sleep or unintended lapses into drowsiness or sleep.” Though brief periods of sleepiness can be normal, it is problematic when it interferes with daily activities and quality of life, manifesting as inappropriate periods of drowsiness. In patients with ES, sleep analysis included polysomnography, Multiple Sleep Latency Test (MSLT) and actigraphy. Diagnoses were established according to the international classification of sleep disorders-third edition (ICSD-3) (Table 1) [22].

**Table 1:** *International Classification of Sleep Disorders, Third Edition*, Diagnostic Criteria

Narcolepsy Type 1 Criteria A and B	Narcolepsy Type 2 All Criteria A-E	Central Hypersomnia due to medical disorder All Criteria A-F
A. Daily periods of irrepressible need to sleep or daytime lapses into sleep, present for at least 3 mo	A. Daily periods of irrepressible need to sleep or daytime lapses into sleep, present for at least 3 mo	A. Daily periods of irrepressible need to sleep or daytime lapses into sleep, present for at least 3 mo
B. Either 1 or 2 or both	B. Mean sleep latency ≤ 8 min and two or more SOREMPs on MSLT. REM within 15 min of sleep onset on the preceding nocturnal polysomnogram may replace one of the SOREMPs.	B. Daytime occurs as consequence of a medical condition.
1. Cataplexy and mean sleep latency ≤ 8 min and two or more SOREMPs on MSLT. REM within 15 min of sleep onset on the preceding nocturnal polysomnogram may replace one of the SOREMPs.	C. No cataplexy	C. The mean latency is ≤ 8 min), and fewer than two SOREMPs on MSLT.
2. Low CSF hypocretin-1 concentration (< 110 pg/mL or less than one-third of control values)	D. CSF hypocretin-1 concentration has not been measured or CSF hypocretin-1 concentration is ≥ 110 pg/mL or greater than one-third of control values.	D. Either 1 or 2 or both:. 1.Mean sleep latency ≤ 8 min on MSLT. 2. Total 24-h sleep time ≥ 660 min on 24-h polysomnographic monitoring or wrist actigraphy (averaged over ≥ 7 d)
	E. The hypersomnolence and/or MSLT findings are not better explained by other causes.	E. Insufficient sleep syndrome is ruled out.
		F. The hypersomnolence and/or MSLT findings are not better explained by other sleep disorder or medications.

*CSF* cerebral spinal fluid, *MSLT* multiple sleep latency test, *REM* rapid eye movement, *SOREMP* sleep-onset rapid eye movement period

PSG was performed with a digital polygraph. Data were collected using an electroencephalogram (EEG) (according to the International 10–20 System), bilateral electrooculogram (E1-M2, E2-M1), electrocardiogram (modified V2 lead), and surface electromyography of the mental and submental muscles. Surface electrodes were placed on both anterior tibialis muscles, masseters, and extensors of fingers. Digital video was recorded with an infrared camera (Sony Ipela., CA) and then synchronized with the PSG data. Respiration was monitored as follows: airflow was measured by a nasal pressure transducer system (AcSleep 119, Biolink Medical, São Paulo, Brazil) and nasal and mouth thermocouple airflow sensor (Pro-Tech Services Inc., Mukilteo, WA); chest and abdominal efforts were measured by respiratory inductive plethysmographic belts (Pro-Tech zRIP module, Pro-Tech Services Inc.); arterial SaO2 was measured by pulse oximetry (Netlink Headbox, Natus Biologic Systems Inc.); snoring sounds were measured using a snoring microphone; body position was determined using a sensor (Netlink Body Sensor Position, Natus Biologic Systems Inc.). All of the technical parameters used were performed in accordance with the AASM Manual for the Scoring of Sleep and Associated Events: Rules, Terminology, and Technical Specification (2007, [23]). The multiple sleep latency test (MSLT) was performed on the day after PSG, with a 5-nap protocol, according to the American Academy of Sleep Medicine guidelines [24].

Actitgraph (*Actitrust*, São Paulo, SP, Brazil) accelerometers were worn on the nondominant wrist continuously for 15 days prior to PSG/MSLT, only taken out during baths, to assess sleep-wakefulness patterns in these patients. Data were stored in the bracelet and transferred by a USB cable to software for analysis. Accelerometer data were stored in 1-minute samples and analyzed for circadian rhythm pattern, total sleep time, sleep latency and number of awakenings.

We performed brain image acquisition using a Siemens Somatom Vision 1.5-T Magnetic Resonance Imaging (MRI) scanner. The device was in regular activity at the University Hospital of Universidade Federal do Ceará under normal operating conditions.

Laboratory tests, including complete blood count (CBC), TSH, T4, blood iron, ferritin, transferrin saturation, liver enzymes, creatinine and electrolytes, were performed in these patients. All patients with excessive sleepiness underwent brain magnetic resonance imaging.

### Subgroup analysis of chronic insomnia

The term chronic insomnia will be used as a disorder with the following diagnostic criteria: (1) difficulty falling asleep, staying asleep or nonrestorative sleep; (2) this difficulty is present despite adequate opportunity and circumstance to sleep; (3) this impairment in sleep is associated with daytime impairment or distress; and (4) this sleep difficulty occurs at least 3 times per week and has been a problem for at least 3 months after COVID infection [22].

### Statistical analysis

Categorical variables were described as absolute frequencies and percentages, while numerical variables were described as averages and standard deviations. Association analyses were performed using Pearson’s chi-square test for categorical variables. Two-tailed *p* values of < 0.05 were considered statistically significant. Statistical analyses were performed with Jamovi®, version 1.0 (The Jamovi Project, n.d.).

### Ethical aspects

The study project was approved by the Research Ethics Committee of Hospital Universitário Walter Cantídio under number 4.092.933. HUWC Research Ethics Committee (CEP) – Rua Coronel Nunes de Melo, 1142 - Rodolfo Teófilo, phone: 3366–8589 (opening hours: 7:00–12:00 h and from 13:00–15:30 h)– E-mail: cephuwc@huwc.ufc.br.

This study complies with the ethical principles of the Declaration of Helsinki. The free and informed consent form was applied. The document was read and informed and agreed by the patient or legal representative, with registration, before the beginning of the protocol, ensuring respect for the patient’s freedom to refuse to participate or to withdraw their consent, at any stage of the research, without no penalty and without prejudice to their care, with, in addition, a guarantee of secrecy to ensure their privacy regarding the confidential data involved in the research. All patients signed an informed consent form, with the right to secrecy and confidentiality of the information obtained, in addition to freedom of refusing to participate in the proposed activities and questions.

## Results

**Fig. 1:**
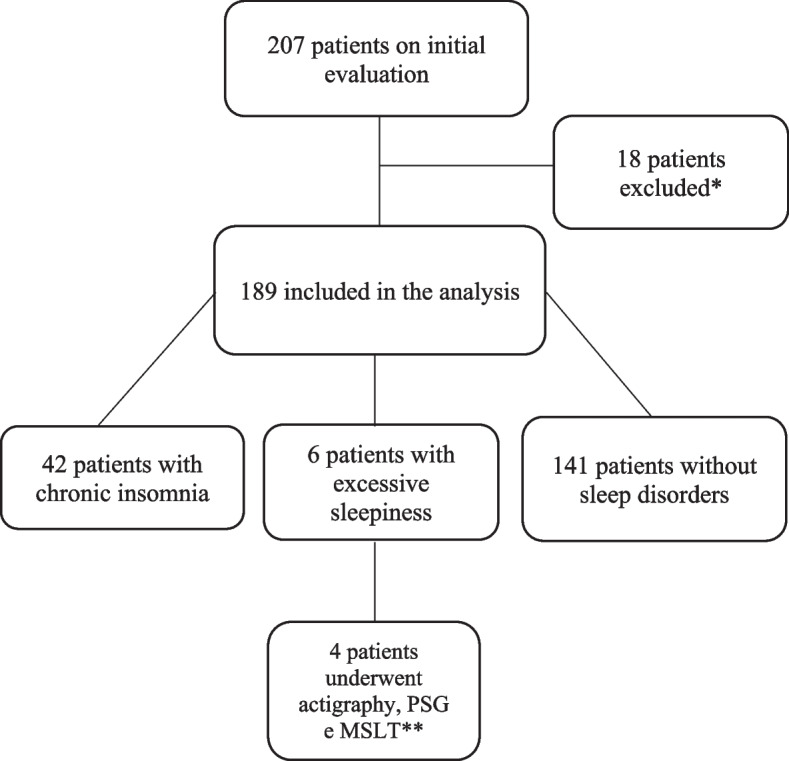
Flowchart of evaluated patients * Eight patients were excluded for lack of RT–PCR confirmation of COVID infection, 8 patients for not having persistent symptoms and 2 for preexisting sleep complaints. **2 patients refused to undergo actigraphy, PSG and MSLT. PSG – polysomnographic; MSLT - multiple sleep latency test

The initial evaluation consisted of 207 patients. Eight patients were excluded for lack of RT–PCR confirmation of COVID infection, eight patients were excluded for not having persistent symptoms, and 3 other patients were excluded for preexisting sleep complaints (Fig. 1).

Among 189 patients included in the long-COVID sample (Table 2), 48 (25.3%) had sleep-related symptoms. Age, sex and COVID severity were similar between patients with sleep disorders and those without. Depression, corticosteroid use and subjective cognitive decline were significantly more common in patients with sleep disorders.

**Table 2:** Sociodemographic and clinical characteristics of patients with long COVID-19, according to sleep symptoms

	No sleep disorder (*n* = 138)	Chronic Insomnia (*n* = 42)	Excessive Sleepiness (*n* = 6)	*p*
Age (sd)	44.9(± 46)	49.7(± 28)	41(± 30)	0.350
Males, n(%)	50 (39)	14(33)	1(13)	0.503
Alcoholism, n(%)	14(11)	9(22)	4(50)	0.068
COVID hospitalization, n(%)	35(28)	13(32)	2(25)	0.856
Corticosteroid use, n(%)	12(9)	17(40)	5(63)	<.001
Headache, n(%)	37(29)	15(36)	4(50)	0.413
Hypo/anosmia, n(%)	37(29)	20(48)	1(13)	0.064
Depression, n(%)	12(9)	12(29)	4(50)	<.001
Anxiety, n(%)	28(22)	12(29)	2(25)	0.565
Cognitve impairment, n(%)				
Dementia, n(%)	10(7.8)	0	0	–
Subjective Cognitive				
Decline, n(%)	63(48.8)	33(78.6)	5(83.3)	0.005

Insomnia was reported by 42 patients (22.2%), and excessive sleepiness (ES) was reported by 6 patients (3.17%). Two of these patients with ES refused to undergo PSG and MSLT. The final subgroup of ES consisted of four patients.

### Clinical and PSG characteristics of patients with ES

Of a total of 4 patients with ES, 3 fulfilled the clinical criteria for central hypersomnia, and one reported symptoms of ES that resolved spontaneously after 6 months without intervention (Table 3) before her first evaluation. Patients with ES also complained of fatigue, inattention and persistent hyposmia.

**Table 3:** Clinical characteristics of four patients with excessive sleepiness after COVID-19 infection

	Case one	Case two	Case three	Case four
Sex	F	M	F	F
Age (years)	35	42	32	55
Time from COVID-19 infection (months)	18	18	13	18
Need for hospitalization	N	N	N	N
Cognitive complaint	Y	Y	Y	Y
Time from ES onset (months)	17	17	12	17
Epworth scale	22	16	21	4
Snoring	N	N	N	N
Fatigue	Y	N	Y	N
Restorative sleep	N	N	N	N
Cataplexy	N	N	N	N
RLS symptoms	N	N	N	N
Treatment	Modafinil 200 mg/d + Methylphenidate 10 mg/d	Modafinil 200 mg/d	Lisdexamphetamine 35 mg/d	–
Improvement of ES	Y	Y	Y	Y

For the majority of patients, symptoms persisted for up to 18.6 weeks (+ 72/−6 mean) after acute infection. The symptoms varied in their prevalence over time.

**Fig. 2:**
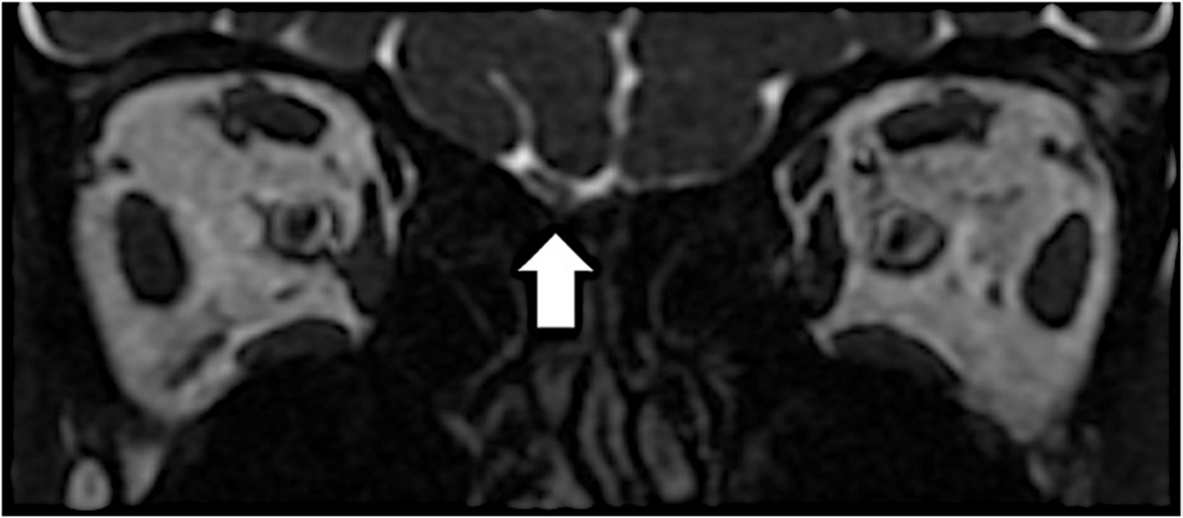
Olfactory bulb atrophy in MRI

Magnetic resonance imaging (MRI) revealed olfactory bulb atrophy in one patient (Fig. 2), while it was normal in the other three. Laboratory results did not reveal abnormalities in any of the patients.

Polysomnographic, MSLT and actigraphic data are described in Table 4. We found two patients with central hypersomnia and one patient with narcolepsy.

**Table 4:** Polysomnographic, MSLT and actigraphic data of four patients with excessive sleepiness after COVID-19 infection

	Case one	Case two	Case three	Case four
**Polysomnographic data**				
Total sleep time (h)	350	380	488	462
Sleep eficiency (%)	87.9	77	94.5	94
WASO (%)	60	44.5	59.3	20
Total microarousal index (number/h)	21.9	11.7	21.4	8.4
Total microarousal	128	97	153	81
N1 latency (min)/% of sleep	0.2/13.7	66/0.8	5.9/0.4	0/6.4
N2 latency (min)/% of sleep	17.7/68.3	88/35.3	7.4/49.6	2/57.5
N3 latency (min)/% of sleep	38/5	102.5/36.7	20.9/25.3	27/53
AHI total (events/h)	7.7	4.9	9.9	8.7
PLMI total (events/h)	0.8	0	0.1	0.1
Atonia in REM sleep	Y	Y	Y	Y
REM latency (min)/% of sleep	240/13	81/27.2	58/24.7	164/21
**MSLT data**				
SOREMPs (number)	0	0	2	0
Mean latency (min)	1.5	5.9	6	–
**Actigraphic data**				
Mean total daily sleep time (hours)	11.6	13.2	7	8.4

*PSG* polysomnographic, *RLS* restless legs syndrome, *WASO* wake after sleep onset, *N1* NREM 1, *N2* NREM 2, *N3* NREM 3, *AHI* apnea hypopnea index, *PLMI* periodic leg movement index, *REM* rapid eye movement, *MSLT* multiple sleep latency test, *SOREMP* sleep onset REM period, *Y* yes, *N* no

After diagnosis, all patients with persistent ES were treated with wakefulness promoters (modafinil in the first case, modafinil and methylphenidate in the second, and lisdexamfetamine in the third). All patients reported improvement with a decrease in Epworth scores and functional improvement.

## Discussion

This study demonstrated a high prevalence of sleep symptoms in a cohort of long-term COVID-19 patients with persistent sleep disorders up to 18 months after infection. Insomnia was the most frequent sleep disorder in these patients. To the best of our knowledge, we have reported the first cases of central hypersomnia after COVID. One of our patients fulfilled clinical and polysomnographic criteria for narcolepsy. All patients with ES responded to wakefulness-promoting agents.

The neuropathological mechanisms in COVID-19 can be related to direct viral invasion, systemic inflammation, neuroinflammation, microvascular thrombosis and neurodegeneration [6, 25]. Autopsy series have demonstrated abnormalities in brain parenchyma and blood vessels possibly related to blood–brain barrier dysfunction leading to neuronal inflammation after hematogenous dissemination [26, 27]. Another possible pathway for the virus to reach the central nervous system (CNS) is viral invasion of the olfactory epithelium and propagation toward the brain through axonal transport [28, 29].

Prolonged latent inflammation, memory T-cell accumulation and decreased ability to respond to new antigens (markers of immunosenescence) might play a role in persistent dysfunction after COVID-19 [26]. Other proposed mechanisms for long COVID include glymphatic system impairment, with glymphatic congestion and reduced clearance of toxins and inflammatory mediators from the CNS [30].

A possible explanation for persistent sleep disorders after COVID-19 might be related to prolonged dysfunction of brainstem nuclei [31]. This dysfunction could be explained by a high concentration of ACE2 receptors (which are used by SARS-CoV-2 to enter cells) in the brainstem [31]. Some of these brainstem nuclei are involved in sleep-wakefulness regulation, such as the dorsal raphe nucleus, pedunculus-pontine nucleus, periaqueductal gray, and laterodorsally tegmental nucleus [31].

Chronic insomnia was the most common sleep disorder after COVID-19 infection, in line with other studies of long COVID-19 patients [32]. Social isolation, stress, anxiety, persistent inflammatory response, and corticosteroid use might justify the high prevalence in our sample. Corticosteroid use was significantly associated with sleep disorders in our cohort. It is possible that changes associated with the acute infection might have precipitated dysfunctional habits, such as excessive caffeine consumption or poor sleep hygiene, which may also contribute to insomnia in a multifactorial mechanism [12].

Anosmia is a common symptom in long COVID-19 patients. A possible mechanism for this association could be invasion of the olfactory epithelium and olfactory nerve with retrograde axonal transport to the hypothalamus via the stria terminalis. One of our patients with excessive sleepiness had olfactory bulb atrophy on MRI, reinforcing a possible association of olfactory nerve invasion and hypothalamic dysfunction resulting in sleep disorders.

Persistent cognitive symptoms were also more common in patients with sleep disturbances in our sample. It is possible that widespread central nervous system damage could result in cognitive dysfunction and sleep disturbances. Neuroinflammation, endothelial dysfunction, systemic inflammation, hypoxia, cerebrovascular disease and the presence of an APOE4 polymorphism might contribute to the coexistence of cognitive and sleep disturbances [32, 33]. Patients with ES could also suffer from impairment of attention, which could impact performance in other cognitive domains.

The association of excessive sleepiness and depression has been previously documented [34]. Depressive symptoms after COVID infection have been reported in up to 22% of patients [12]. In our cohort, depression was more common in patients with sleep disorders, reaching 50% in patients with ES. Depression in COVID patients might be associated with social isolation, uncertainties about the future, systemic inflammatory response and disability associated with long-term COVID symptoms and sequelae. The relationship between ES and depression is probably bidirectional, with poor sleep quality negatively affecting mood state and poor sleep quality and ES as a possible result of depression [12, 32].

We have reported three patients with documented central hypersomnia after COVID-19. One of them fulfilled the criteria for narcolepsy. All 3 patients were young and had no sleep disorders prior to COVID infection, suggesting a strong association with SARS-CoV-2 infection. To our knowledge, this is the first report of this association in the literature. Similar to other neurotropic viruses, severe acute respiratory syndrome coronavirus 2 (SARS-CoV-2) may be the culprit for instigating the relapse of Klein Levin Syndrome, in a young patient, with no other precipitating factors [35].

During the H1N1 influenza pandemic of 2009, an increased incidence of narcolepsy was reported in China and Europe [36]. Large-scale vaccination for H1N1 was also associated with an increased risk of narcolepsy in children and young adults, particularly the Pandemrix vaccine (GlaxoSmithKline, Dresden, Germany) [37]. Narcolepsy was hypothesized to be the result of molecular mimicry, with CD4+ T cells cross-reactive to hypocretin fragments leading to hypothalamic damage [38].

In addition to the possibility of direct viral infection, hypersomnia after COVID might be related to immune and inflammatory hyperactivation resulting in hypothalamic infiltration by CD4+ and CD8+ T cells leading to neuronal damage [29]. There is also the possibility of molecular mimicry between viral antigens and hypocretin fragments leading to an immune response against hypocretinergic cells, similar to what has been described with H1N1 influenza infections [38].

Responses to treatment with wakefulness-promoting agents were observed in all cases, suggesting a similar response profile to other patients with central hypersomnia. On the other hand, 2 patients (50%) had self-limited symptoms that resolved after 6 months in one case and 15 months in another, implying reversible dysfunction of the sleep-wakefulness cycle in some cases.

The present study has some limitations. An initial selection bias resulted from a higher probability of patients with more severe symptoms participating in our study. Unfortunately, we were not able to perform PSG and MSLT and dose CSF hypocretin levels in all patients with sleep-related symptoms due to financial limitations. A few patients with ES did not undergo PSG and MSLT due to lack of consent. Nevertheless, we believe these limitations do not impact the clinical findings that expand central hypersomnia as a possible spectrum of long-onset COVID syndrome.

## Conclusion

Sleep disorders are common in patients with long COVID syndrome, particularly insomnia. Chronic insomnia and central hypersomnia may expand the spectrum of post-COVID sleep disturbances. These patients seem to respond to the wakefulness promoter and may have a self-limited course in some cases. This study underscores the importance of sleep disorders in post-COVID patients. Further studies should address the possible pathologic mechanisms and clinical course of these conditions to understand their prognosis and guide their management.

## Data Availability

All of the material is owned by the authors and no permissions are required. All of the material are available for publication. To consult and check the data of this study, contact alisamoura@gmail.com or pbraganeto@ufc.br

## Abbreviations

AASM: American Academy of Sleep Medicine
ACE2: Angiotensin Converting Enzyme 2
AHI: Apnea hypopnea index
APOE4: Apolipoprotein E4
CA: California
Cognitive Examination–Revised: Cognitive Examination–Revised
*CBC*: Complete blood count
TCD4: Thymus-derived lymphocytes CD4
TCD8: Thymus-derived lymphocytes CD8
*CNS Central*: Central nervous system
*CSF*: Cerebrospinal fluid
*COVID-19*: Corona Virus Disease 2019 caused by SARS-CoV-2
*DSM-V*: *Diagnostic and Statistical Manual of Mental Disorders (Fifth Edition)*
EEG: Electroencephalogram
ES: Excessive sleepiness
H1N1: Influenza A
MERS: Middle East Respiratory Syndrome
MMSE: Mini-Mental State Examination
*MRI*: Magnetic resonance imaging
*MSLT*: Multiple sleep latency test
*NREM*: Non–rapid eye movement
*PLMI*: Periodic leg movement index
PSG: Polysomnography
*REM*: Rapid eye movement
*RLS*: Restless legs syndrome
RT-PCR: Reverse transcription polymerase chain reaction (this is the test used to detect a specific RNA)
SARS: Severe Acute Respiratory Syndrome
SARS-COV-2: Severe Acute Respiratory Syndrome coronavirus 2
*SOREMP*: Sleep-onset rapid eye movement period.
T4: Thyroxine
TSH: *Thyroid Stimulating Hormon*
WA: *Washington*
WASO: Wake after sleep onset
